# The Breath

**Published:** 1894-01

**Authors:** J. Taft


ARTICLE V.

### THE BREATH.

BY DR. J. TAFT.

Impure breath is liable to occur at all periods of life. It is found in a great variety of phases—from the slightest perceptible deviation from the normal state to that in which it is loaded with a large amount and great variety of excrementitious matter from various sources, perceived sometimes even by the sense of taste, at least so far as the patient is concerned, but more especially by the olfactories making such an impression through this channel as to produce nausea, and, indeed, the infection of contagious diseases may be carried from one person to another by the breath. A vitiated breath as a means of conveying the seeds of diseases is great.

We involuntarily turn away from the fetid breath of even our best friends. One may be beautiful in face and form, attractive in manner, and fascinating in conversation, and possess personal magnetism that may be well-nigh irresistible, and yet an offensive breath will neutralize the influence of all these qualities.

In no other relation is the subject of greater moment than that existing between the dentist and his patient.

The degree of offensiveness is modified by the acuteness of the olfactories of one or both parties, together with the conditions of the exhalations, and the course or sources of the fetid breath.

If the dentist is subjected for a considerable time to this condition of things, his general health may become affected in a more or less marked degree, according to his own susceptibility, and to the degree of vitiation of the air he is compelled to inhale. There can hardly be a doubt but what many suffer very greatly from this cause; and though one may not suffer much, or apparently at all in this respect, yet a sense of discomfort and uneasiness is always experienced by the dentist in his professional work when subjected to a vitiated atmosphere, and this will necessarily interfere somewhat with the thoroughness of his operations. It is a serious question whether the dentist ought ever to subject himself to such embarrassments, at least for more than a few minutes at a time. It certainly would be for the welfare and interest of both the patient and the dentist, were the former to be put on proper treatment before having extended operations made on the teeth.

The offensive breath of the operator is a matter of special interest to the patient as well as to himself. The dentist has no right to make himself, or even to be a nuisance in any respect to those whom he serves, and if he has any just appreciation of the fitness of things, he will see to it that his presence in every respect is rendered as little objectionable as possible.

The patient is not likely to suffer ill-health or prolonged discomfort from the fetid breath of the dentist, and, indeed, many of less acute sensibility will be inclined by and by to seek more acceptable service.

The dentist should be able at all times to discriminate in regard to the character of his own breath; it may sometimes be a necessity to submit to this annoyance from others, but he should see to it that he never imposes on his patients in this way. I have known superior operators —persons of gentlemanly deportment in every other respect—whose breath was in such a condition as to disgust all those who came within its influence; indeed, I have known some such who were compelled to abandon the practice of dentistry.

There are some instances in which this affection seems uncontrollable, but generally it is amenable to proper treatment, and can either be modified, masked, or wholly eradicated.

Derangement of the stomach, alimentary tract, kidneys, liver or skin is almost certain to result in more or less marked change of the breath, from the fact that in part, at least, the waste that thus fails to be removed is thrown into the lungs, and will, in many instances, produce a markedly offensive breath. But in some instances the breath may be contaminated with excrementitious matter that possesses little or no offensive odor. The defective function of the digestive apparatus is, in nearly all cases, a source of fetid breath. Disease of the lungs of almost every variety is attended with more or less vitiation of the breath.

Of the local sources of this difficulty there are many, and of these there may be said to be two classes, the one embracing all the local disorders that may contaminate the breath after it leaves the lung; this will embrace the various forms of diseases found in the throat, mouth and nose. Diphtheria, scarlet fever, tonsilitis, and, perhaps, some other affections, though affecting the entire system, possess a local manifestation that results in greatly vitiated breath.

The various catarrhal affections that are found in the nose, the throat and mouth, in all cases more or less affect the breath, ranging all the way from the very mild, almost imperceptible change, to an intolerably disgusting degree. This affection should be well studied by the dentist, in order that he may be able to give his patient some, if not permanent, relief, and that he may protect himself so far as he may against an intensely annoying and offensive condition.

Diseases of the gum and mucous membrane of the jaws are often the occasion of this offensive condition. This will result, sometimes, from a vitiated exudate from the mucous membrane, or it may occur, as is frequently the case, from a discharge from the margins of the gums, and from the sockets of the teeth.

Necrosis and sloughing of the bony tissues of the sockets nearly always produce a very offensive condition.

The discharge from alveolar abscess is oftentimes so so vitiated as to load the breath which passes out of the mouth with an exceedingly offensive odor.

Decayed teeth are charged, especially by various medical writers, as a very frequent cause of offensive breath. This is true not only of physicians, but to a greater extent, perhaps, of the laity. There is not, however, as much in this as is usually attributed to it. Occasionally cases are presented in which an exceedingly foul breath is wholly attributed to one small innocent cavity of decay in the grinding surface of a molar tooth. Were all other causes of offensive breath eliminated, than that which comes directly from the decay of the teeth, there would be, in the aggregate, an immense improvement.

Another fruitful source of offensive odors of the oral cavity is found in the presence of foreign substances, or matter in the mouth in the shape of soft salivary calculus, accumulation of food, and a glutinated mucous deposited on the teeth or the artificial dentures undergoing decomposition, and necessarily throwing off an effluvia that will be mixed with the breath. The saliva and mucus, mixed thus with foreign substance, and retained for an undue time in the mouth, will undergo such change as to present a very offensive condition. Now as to these extraneous causes of the affection under consideration, it is not difficult for the educated patient to determine what should be done; simply purification of the oral cavity in the most thorough manner by the entire removal of all offensive material; and after this the intelligent use of disinfectants on the teeth, mucous membrane and dental plates, if they are in the mouth.

With a large variety of disinfectants, antiseptics, cleansing materials and methods there is no difficulty in rendering almost every such case free from the objectionable condition, for a time at least sufficient for operations.

A great many formulas have been given for the correction of offensive breath; the suggestions made for the use of these, however, in the majority of cases, are on a false basis; with many of them it is only the substitution of one odor for another, or the mixing of two offensive conditions, and producing a third that is, perhaps, temporarily more tolerable than either of the others.

In treatment here, however, the aim should be, as in all other medical treatment, to attain the most permanent results; that, doubtless, is the true theory of all medical and surgical practice.

Temporizing should never be employed when something better can be attained.

It is very desirable that the profession should give more attention to this subject than hitherto. In our literature very little will be found on this subject, and in all medical literature, so far as I have been able to examine, only a fugitive reference to it has been here and there made; and I may here refer those who have not investigated the subject to a little work entitled "The Breath and the Disorders which give it a Fetid Odor," by Dr. Joseph W. Howe, the third edition of which was issued in 1885, and a paper published in the *Dental Register*, by Dr. D. C. Hawxhurst, vol. xxvii, page 104.—*Ohio Journal.*

